# Supplementary material for: Complex hemolymph circulation patterns in grasshopper wings
Source: Commun Biol. 2023 Mar 23;6:313. doi: 10.1038/s42003-023-04651-2 (PMC10036482; doi:10.1038/s42003-023-04651-2)
Supplement: Supplementary file 2 — Supplementary Material [file 42003_2023_4651_MOESM2_ESM.pdf]

Supplementary Material:
Data videos were recorded at the Harvard Center for Biological Imaging by MK Salcedo between July and December 2017. This research collection was supported by the Mahadevan and Holbrook labs.

Movies were annotated and edited in Fiji. Original .czi files (Carl Zeiss Images) were opened and processed in Fiji using the Bioformats Plugin. Analysis code and tracking data can be found: https://github.com/maryksalcedo/wingflow_grasshoppers.git.

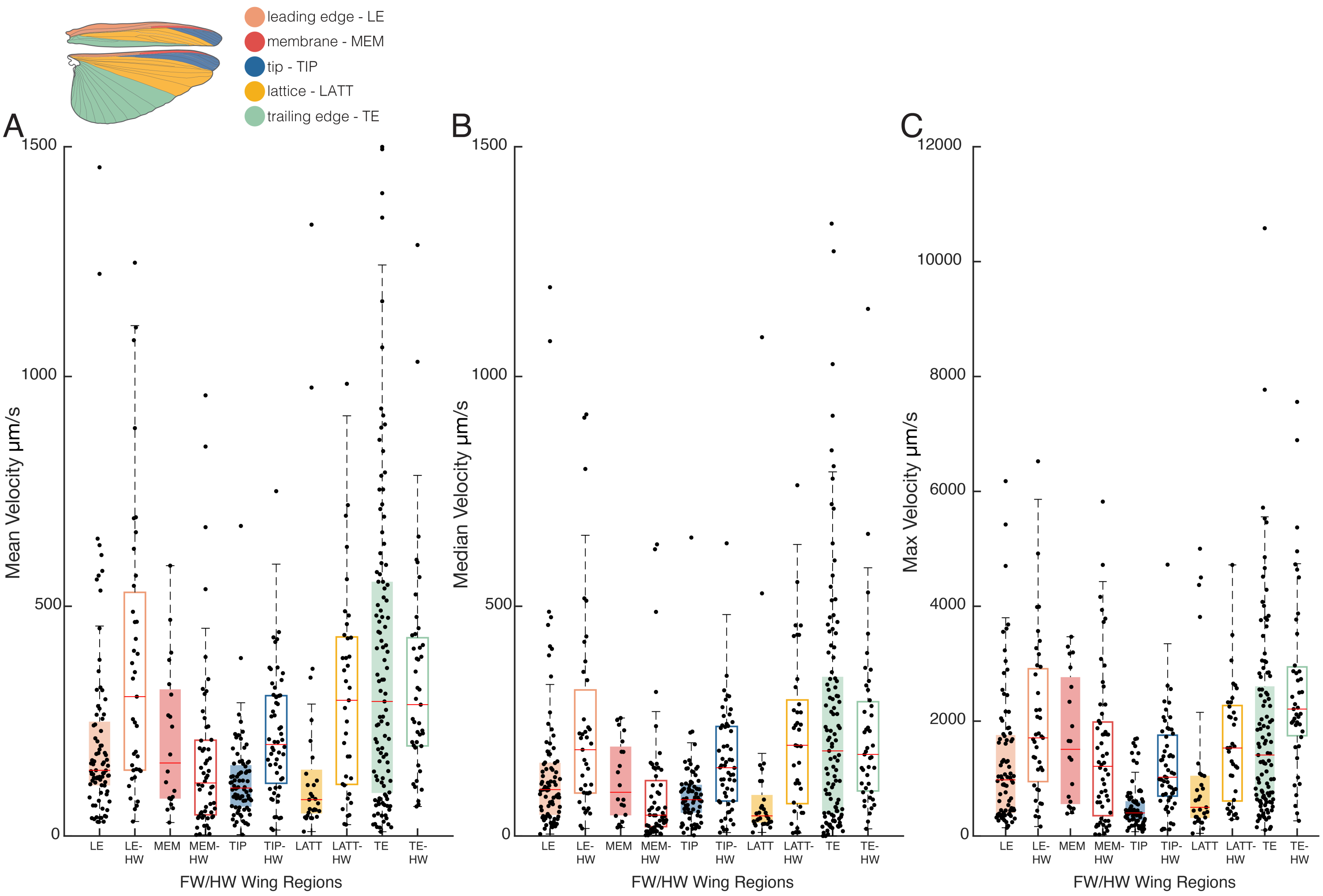

Supplementary Figure 1. Mean, median, and maximum instantaneous velocities across wing regions. The three above plots describe velocities of approximately 500 particles tracked across 8 insects. Calculation and tracking methods are described in the main paper. For each box, the central mark is the median, and the bottom/top edges indicate 25th and 75th percentiles. Whiskers extend to extreme data points.
