# Supplementary material for: Complex hemolymph circulation patterns in grasshopper wings
Source: Commun Biol. 2023 Mar 23;6:313. doi: 10.1038/s42003-023-04651-2 (PMC10036482; doi:10.1038/s42003-023-04651-2)
Supplement: Supplementary file 3 — Description of Additional Supplementary Files [file 42003_2023_4651_MOESM3_ESM.pdf]

Description of Additional Supplementary Files

File name: Supplementary Movie 1
Description: Left view at the wing base of the forewing, shows that particles and hemocytes move together with pulsatile flow, into the wing. Flow here is considered leaky since it is not constrained to tubular veins at this point in the wing. Right view, this video shows flow in the costa vein of the hindwing but also flow returning to the body through the trailing edge of the forewing.

File name: Supplementary Movie 2
Description: Left view of both the leading edge and membrane regions showing pulsatility of flow in the leading edges drives flow into the membrane region. Right view of both the leading edge and membrane regions showing pulsatile flow in the leading edge veins and leaky flow in the membrane region. Particles can cycle in between the tubular vein and membrane sinus.

File name: Supplementary Movie 3
Description: Left view of hindwing membrane region. Particles in membrane flow readily from vein to membrane. Strong pulsatility drives flow to the wing base. Nerve branches are visible and tracheal compressions/decompressions occur (as noted) with pulses.

File name: Supplementary Movie 4
Description: Left view of forewing wing tip showing continuous particle movement down the chord of the wing. Nerve sensilla and branches (faint white lines) are visible. Right view of wing tip, but also showing the membrane and leading edges of the hindwing. Flow moves from pulsatile leading edge to more continuous flow down the chord of the wing.

File name: Supplementary Movie 5
Description: Left view of forewing lattice region showing both continuous flow and bi-directional flow. With bi-directional flow, flow directions can meet each other and then reverse. Right view of the hindwing lattice region where vein junctions are primarily at right angles to each other and flow traverses across a mountain-valley fold pattern (the hindwing is a corrugated fan). Flow moves continuously before rerouting to body in the the annal veins.

File name: Supplementary Movie 6
Description: Left view of trailing edge of the forewing where pulsatility returns as flow is being sucked back into the body. Middle view of the annal veins of the hindwing, which make up the trailing edge region. Particles become more pulsatile as they are pulled into the auxillary cord (posterior scutellar branch) which acts as a return conduit into the posterior thoracic wing heart. Right view of the hindwing trailing edge, specifically the wing edge. Even in the smallest veins at the edge of the wing, flow occurs.

File name: Supplementary Movie 7
Description: View of the dorsal thorax with all four wings spread. Pictorial hearts mark the locations of thoracic wing hearts and blue arrows indicate the return conduits into those hearts. Note that air sacs in between the forewing and hindwing affect flow rates, but that is unquantified.

File name: Supplementary Movie 8
Description: View of the thoracic wing heart that is more anterior and responsible for pulling hemolymph out of the forewing. The anterior scutellar branch acts as a “return conduit” for flow

from the forewing, as the anterior thoracic heart, pulls hemolymph from the wing. There are clear, distinct pulses in the tissues.
