# Supplementary material for: Complex hemolymph circulation patterns in grasshopper wings
Source: Commun Biol. 2023 Mar 23;6:313. doi: 10.1038/s42003-023-04651-2 (PMC10036482; doi:10.1038/s42003-023-04651-2)
Supplement: Supplementary file 12 — Reporting Summary [file 42003_2023_4651_MOESM12_ESM.pdf]

nature portfolio

Corresponding author(s): Mary Salcedo

Last updated by author(s): Jan 30, 2023

Reporting Summary

Nature Portfolio wishes to improve the reproducibility of the work that we publish. This form provides structure for consistency and transparency in reporting. For further information on Nature Portfolio policies, see our Editorial Policies and the Editorial Policy Checklist.

Statistics

For all statistical analyses, confirm that the following items are present in the figure legend, table legend, main text, or Methods section.

n/a	Confirmed	
☐	☒	The exact sample size (n) for each experimental group/condition, given as a discrete number and unit of measurement
☐	☒	A statement on whether measurements were taken from distinct samples or whether the same sample was measured repeatedly
☐	☒	The statistical test(s) used AND whether they are one- or two-sided<br>*Only common tests should be described solely by name; describe more complex techniques in the Methods section.*
☒	☐	A description of all covariates tested
☒	☐	A description of any assumptions or corrections, such as tests of normality and adjustment for multiple comparisons
☒	☐	A full description of the statistical parameters including central tendency (e.g. means) or other basic estimates (e.g. regression coefficient) AND variation (e.g. standard deviation) or associated estimates of uncertainty (e.g. confidence intervals)
☒	☐	For null hypothesis testing, the test statistic (e.g. F, t, r) with confidence intervals, effect sizes, degrees of freedom and P value noted<br>*Give P values as exact values whenever suitable.*
☒	☐	For Bayesian analysis, information on the choice of priors and Markov chain Monte Carlo settings
☒	☐	For hierarchical and complex designs, identification of the appropriate level for tests and full reporting of outcomes
☐	☐	Estimates of effect sizes (e.g. Cohen's d, Pearson's r), indicating how they were calculated

Our web collection on statistics for biologists contains articles on many of the points above.

Software and code

Policy information about availability of computer code

Data collection	Particle movement was captured in 8 adult Schistocera americana (approx. 3-5 months old) on a fluorescent microscope (Zeiss AxioZoom V16 Zoom, using Zeiss software) at the Harvard Center for Biological Imaging (Cambridge, MA).
Data analysis	Code used in data analysis was previously written and is cited in the main text as well as here (1-4). Matlab analysis code can be found at: https://github.com/maryksalcedo/wingflow_grasshoppers.git. (1) Cardwell, N.D., Vlachos, P.P. and Thole, K.A., 2011. A multi-parametric particle-pairing algorithm for particle tracking in single and multiphase flows. Measurement Science and Technology, 22(10), p.105406. (2) Guo, T., Ardekani, A.M. and Vlachos, P.P., 2019. Microscale, scanning defocusing volumetric particle-tracking velocimetry. Experiments in Fluids, 60, pp.1-14. (3) Jun, B.H., Guo, T., Libring, S., Chanda, M.K., Paez, J.S., Shinde, A., Wendt, M.K., Vlachos, P.P. and Solorio, L., 2020. Fibronectin-expressing mesenchymal tumor cells promote breast cancer metastasis. Cancers, 12(9), p.2553. (4) Jun, B.H., Ahmadzadegan, A., Ardekani, A.M., Solorio, L. and Vlachos, P.P., 2022. Multi-feature-Based Robust Cell Tracking. Annals of Biomedical Engineering, pp.1-14.

For manuscripts utilizing custom algorithms or software that are central to the research but not yet described in published literature, software must be made available to editors and reviewers. We strongly encourage code deposition in a community repository (e.g. GitHub). See the Nature Portfolio guidelines for submitting code & software for further information.

Data

Policy information about availability of data

All manuscripts must include a data availability statement. This statement should provide the following information, where applicable:

- Accession codes, unique identifiers, or web links for publicly available datasets
- A description of any restrictions on data availability
- For clinical datasets or third party data, please ensure that the statement adheres to our policy

Code used in data analysis was previously written by Cardwell et al. (2011), Guo et al (2019), Jun et al. (2020), and Jun et al. (2022). Matlab analysis code can be found at https://github.com/maryksalcedo/wingflow_grasshoppers.git.

Human research participants

Policy information about studies involving human research participants and Sex and Gender in Research.

Reporting on sex and gender	*Use the terms sex (biological attribute) and gender (shaped by social and cultural circumstances) carefully in order to avoid confusing both terms. Indicate if findings apply to only one sex or gender; describe whether sex and gender were considered in study design whether sex and/or gender was determined based on self-reporting or assigned and methods used. Provide in the source data disaggregated sex and gender data where this information has been collected, and consent has been obtained for sharing of individual-level data; provide overall numbers in this Reporting Summary. Please state if this information has not been collected. Report sex- and gender-based analyses where performed, justify reasons for lack of sex- and gender-based analysis.*
Population characteristics	*Describe the covariate-relevant population characteristics of the human research participants (e.g. age, genotypic information, past and current diagnosis and treatment categories). If you filled out the behavioural & social sciences study design questions and have nothing to add here, write "See above."*
Recruitment	*Describe how participants were recruited. Outline any potential self-selection bias or other biases that may be present and how these are likely to impact results.*
Ethics oversight	*Identify the organization(s) that approved the study protocol.*

Note that full information on the approval of the study protocol must also be provided in the manuscript.

Field-specific reporting

Please select the one below that is the best fit for your research. If you are not sure, read the appropriate sections before making your selection.

☒ Life sciences ☐ Behavioural & social sciences ☐ Ecological, evolutionary & environmental sciences

For a reference copy of the document with all sections, see nature.com/documents/nr-reporting-summary-flat.pdf

Life sciences study design

All studies must disclose on these points even when the disclosure is negative.

Sample size	Adults of Schistocerca americana were used. No sample-size calculation was performed. Eight adults were chosen based on wing appearance. Over 800 particle trajectories were measured across all the adults. We felt these particle trajectories accurately represented circulation trends in these adult insects.
Data exclusions	Data was excluded if particle trajectories were tracked for less than 25 data points. The exclusion criteria was not previously established but examined as data was being analyzed.
Replication	These experiments are a type of replication from Chintapalli and Hillyer's 2016 work with fluorescent beads in mosquitoes. The experiment has been verified numerous times in grasshoppers and in further work of Salcedo's with dragonflies (not published).
Randomization	Individuals were randomly chosen from a healthy colony of several hundred Schistocera and examined for healthy, untattered wings.
Blinding	Blinding was not relevant to this study as individuals were examined separately under all the same conditions.

Reporting for specific materials, systems and methods

We require information from authors about some types of materials, experimental systems and methods used in many studies. Here, indicate whether each material, system or method listed is relevant to your study. If you are not sure if a list item applies to your research, read the appropriate section before selecting a response.

Materials & experimental systems

n/a	Involved in the study
☒	☐ Antibodies
☒	☐ Eukaryotic cell lines
☒	☐ Palaeontology and archaeology
☐	☒ Animals and other organisms
☒	☐ Clinical data
☒	☐ Dual use research of concern

Methods

n/a	Involved in the study
☒	☐ ChIP-seq
☒	☐ Flow cytometry
☒	☐ MRI-based neuroimaging

Animals and other research organisms

Policy information about studies involving animals; ARRIVE guidelines recommended for reporting animal research, and Sex and Gender in Research

Laboratory animals	Schistocerca americana nymphs (2nd–4th instars) obtained from USDA (Sydney, Montana) and allowed to eclose in lab. Adults (3-5 months old) were used in the experiment.
Wild animals	Wild animals were not used in this study.
Reporting on sex	Sex was not considered in this study and has not been collected. The same general circulation trends were observed in all individuals.
Field-collected samples	Schistocerca americana nymphs (2nd–4th instars) obtained from USDA (Sydney, Montana) were maintained at 30–35 C (16:8 hr light cycle) and reared in accordance with USDA Aphis permits (#:P526P-16-04590).Once nymphs eclosed, adults were placed in a separate enclosure. Adults were regularly fed romaine lettuce, which supplied both nutrition and water.
Ethics oversight	Study was performed under USDA Aphis permits (#:P526P-16-04590). No further ethical approval was required.

Note that full information on the approval of the study protocol must also be provided in the manuscript.
